# *PIK3CA* mutations associated with a poor postoperative prognosis in patients with pulmonary pleomorphic carcinoma: a retrospective cohort study

**DOI:** 10.1186/s12885-022-10176-4

**Published:** 2022-10-15

**Authors:** Kensuke Kojima, Saki Imai, Hironobu Samejima, Ayako Fujiwara, Toshiteru Tokunaga, Hyungeun Yoon, Kyoichi Okishio

**Affiliations:** 1 Department of General Thoracic Surgery, National Hospital Organization Kinki-Chuo Chest Medical Center, 1180 Nagasone-cho, Kita-ku, Sakai-shi, Osaka, 591-8555 Japan; 2 Clinical Research Center, National Hospital Organization Kinki-Chuo Chest Medical Center, Osaka, Japan; 3 Department of Thoracic Oncology, National Hospital Organization Kinki-Chuo Chest Medical Center, Osaka, Japan

**Keywords:** Multivariable cox proportional analysis, Overall survival, *PIK3CA* mutation, Pulmonary pleomorphic carcinoma

## Abstract

**Background:**

Pulmonary pleomorphic carcinoma (PPC) is a rare type of non-small cell lung cancer characterized by high malignancy and a poor prognosis. PPC is associated with a high frequency of postoperative relapse, and shows resistance to chemotherapy. The high malignancy of cancers is associated with genomic instability, which is related to mutations of tumor suppressor genes, such as tumor protein p53 (*TP53*) and ataxia-telangiectasia mutated (*ATM*). In addition, signaling pathways involving the oncogenes such as phosphatidylinositol-4,5-bisphosphate 3-kinase catalytic subunit alpha (*PIK3CA*) and epidermal growth factor receptor (*EGFR*) are associated with resistance to chemotherapy. However, the association of PPC with these gene mutations remains unknown. We investigated the impact of *TP53*, *ATM*, *PIK3CA*, and *EGFR* mutations on the postoperative prognosis of PPC.

**Methods:**

Fifty-five patients with PPC who underwent complete resection were studied. A gene mutation analysis was performed using next-generation sequencing. Postoperative overall survival of patients with gene mutations was evaluated using a multivariable Cox proportional hazards model in which the explanatory variables were the presence of each gene mutation, and the confounding factors were pathological stage and age. The robustness of the results was evaluated by a sensitivity analysis.

**Results:**

The frequencies of pathogenic mutations in *TP53*, *ATM*, *PIK3CA*, and *EGFR* were 47, 0, 7, and 9%, respectively. A multivariable analysis adjusted for pathological stage and age showed a significant difference for only *PIK3CA* mutations. The hazard ratio (HR) for overall survival in cases with pathogenic mutations of *PIK3CA* for wild type or non-pathogenic mutations was 4.5 (95% confidence interval [CI] 1.1–18.8). Likewise, sensitivity analyses adjusted for pathological stage and sex (HR, 7.5; 95% CI 1.7–32.4) and for age and sex (HR, 5.4; 95% CI 1.4–21.7) resulted in similar findings. Although three patients with pathogenic mutations of *PIK3CA* that recurred postoperatively were treated by chemotherapy or immunotherapy, they survived for less than 2 years.

**Conclusions:**

The postoperative prognosis of PPC with *PIK3CA* pathogenic mutations is particularly poor. Pathogenic mutations of *PIK3CA* may be a postoperative prognostic marker. Inhibition of signaling pathways associated with *PIK3CA* mutations may also be a target for chemotherapy after relapse of PPC.

**Supplementary Information:**

The online version contains supplementary material available at 10.1186/s12885-022-10176-4.

## Background

Pulmonary pleomorphic carcinoma (PPC) is a rare histological type of non-small cell lung cancer (NSCLC) that accounts for approximately 0.4% of all cases of lung cancer [1]. Pathologically, it is defined as adenocarcinoma, squamous cell carcinoma, or large cell carcinoma, containing a component of spindle or giant cells with a sarcomatoid tumor component of at least 10% [2]. Although surgical resection is the first-line treatment for PPC as well as other histological types of lung cancer, postoperative relapse and metastasis occur frequently because of the aggressive progression [3, 4]. Treatment for postoperative relapse is often difficult due to resistance to radiotherapy and chemotherapy, which are characteristics of PPC [5]. Therefore, the establishment of a treatment strategy for this disease is highly desirable.

Recently, the knowledge of cancer-related genes in lung cancer has been accumulated thorough the development of next-generation sequencing technology [6], and it is expected that the precision medicine will be established for future lung cancer treatment. The tumor protein p53 (*TP53*)—a tumor suppressor gene that regulates the cell cycle and apoptosis—is known to be the most mutated gene in human cancers [7, 8]. A high frequency of *TP53* mutations has also been reported in PPC [9]. Ataxia-telangiectasia mutated gene (*ATM*) is known as a tumor suppressor gene that functions to regulate the cell cycle and repair damaged DNA. In recent years, many studies have reported that *ATM* mutations are involved in the development of lung cancer [10, 11]. The phosphatidylinositol-4,5-bisphosphate 3-kinase catalytic subunit alpha gene (*PIK3CA*) encodes the major catalytic subunit of the target protein—the phosphatidylinositol 3-kinases (PI3K)—and is an oncogene that contributes to key signaling, which is required for a wide range of normal cellular functions [12]. Epidermal growth factor receptor (EGFR)-tyrosine kinase inhibitors (TKIs), which inhibit the function of the mutated products of *EGFR*—a driver gene in NSCLC—have been demonstrated to improve the prognosis and are widely used for NSCLC in clinical practice [13]. There are also reports that *PIK3CA* mutations are associated with the acquisition of resistance to EGFR-TKIs [14, 15].

However, due to its rarity, little is known about how *TP53*, *ATM*, *PIK3CA*, and *EGFR* mutations in PPC affect the clinical prognosis after surgery. This information may provide one strategy for the effective treatment of PPC harboring therapeutic resistance. We performed a genetic analysis of surgically resected tissue of PPC using next-generation sequencing and identified *TP53*, *ATM*, *PIK3CA*, and *EGFR* mutations. We then used multivariable analyses to evaluate the association of these genetic mutations with the postoperative prognosis of patients with PPC.

## Methods

### Patients

We retrospectively studied patients who underwent complete resection of NSCLC at the National Hospital Organization Kinki-Chuo Chest Medical Center (KCMC) between February 2002 and January 2021, and selected 67 patients with a pathological diagnosis of PPC. We defined complete resection as a grossly or microscopically removed tumor, which corresponds to R0 in the residual tumor (R) classification. The histopathological diagnosis according to the current 2015 World Health Organization classification was performed by pathologists belonging to KCMC [16]. Medical information, including age, sex, smoking status, surgical procedure, pathological tumor-node-metastasis (TNM) classification (eighth edition), PD-L1 expression status, and the outcome after surgery were collected from medical records. The present study was approved by the Institutional Review Board of KCMC (Approval number: 2020–067). The cut-off date for the analysis was set at June 30, 2022. The Institutional Review Board of KCMC waived the requirement for informed consent from all research participants due to the retrospective and anonymous nature of this study. Information about opting out of the study was provided on the homepage of KCMC. All methods were in accordance with relevant guidelines and regulations.

### PD-L1 immunohistochemistry

We evaluated all viable cancer cells in the entire pathological tissue section of each tumor sample. The PD-L1 clone 22C3 pharmDx kit and Dako Automated Link 48 platform (Agilent Technologies, Dako, Carpinteria, CA, USA) were used to examine the PD-L1 expression. We calculated the PD-L1 tumor proportion score (TPS) as the percentage of complete or partial membrane staining in a sample. The cut-off values for the expression of PD-L1 were set at 50 and 1% based on a previous clinical study. We categorized the tumor samples of each patient into 3 groups (< 1% [negative], 1–49% [low expression], and ≥ 50% [high expression]) based on the presence of positively stained cells in specimen.

### Genomic DNA extraction and quality check

To investigate alterations in cancer-related genes, we obtained 67 lung tissues specimens from patients with PPC by surgical resection or trans-bronchial lung biopsy. All samples were fixed by formalin and embedded in paraffin (FFPE). Genomic DNA (gDNA) was extracted from FFPE samples using QIAamp DNA FFPE Tissue Kit (QIAGEN, Japan) according to manufacturer’s protocol. After extraction, the DNA concentration was quantified by Qubit® 3.0 Fluorometer (Thermo Fisher Scientific, USA). The gDNA quality was determined by the A_260_/A_280_ and A_260_/A_230_ ratios using NanoDrop 1000 (Thermo Fisher Scientific). We excluded 8 of the 67 samples due to failure to extract a sufficient amount of DNA.

### Library preparation and sequencing with the Cancer HotSpot panel

DNA libraries were generated by PCR using AmpliSeq for Illumina Cancer HotSpot Panel v2 (Illimina, USA) in 59 samples. AmpliSeq for Illumina Cancer HotSpot Panel v2 targets 2800 mutations in 50 cancer-related genes, including the genes of interest—*TP53*, *ATM*, *PIK3CA* and *EGFR*—in our study. gDNA (20 ng) was used to amplify the panel range of genes. The libraries were amplified using AmpliSeq Library PLUS for Illumina (Illumina, USA) according to manufacturer’s instructions. Each library was quantified using an Agilent DNA 100 kit with an Agilent 2100 Bioanalyzer (Agilent Technologies, USA). Sequencing was performed using a MiSeq NGS system (Illumina, USA). The MiSeq sequence data was processed and analyzed using BaseSpace Sequence Hub. Of the 59 samples, we excluded one sample because it was impossible to analyze due to an error. In comparison to the UCSC hg19 reference genome, sequences with amino acid changes were identified as somatic mutations.

### Overall survival and relapse-free survival

The primary outcome of this study was overall survival (OS) after surgery, defined as the time from the date of curative resection to the date of death by any cause or the date of the last follow-up examination. The secondary outcome was relapse-free survival (RFS), which was defined as the length of time for which the patient survived after curative resection (as the primary treatment) without any signs or symptoms of cancer or death from any cause.

### Statistical analyses

Finally, 55 patients were included in the statistical analysis (three patients of 58 patients in whom a DNA analysis of PPC was performed were excluded because we were unable to obtain medical information). The probability of OS was assessed using the Kaplan–Meier method and log-rank tests. A multivariable Cox proportional hazards analysis was performed to estimate the hazard ratios (HRs), with adjustment by risk factors for mortality or relapse. In a multivariable Cox proportional hazards analysis, the number of covariates that be can analyzed is the number of cases with a particular outcome ÷ 10. In other words, a multivariable Cox proportional hazards model requires a minimum of 10 outcome events per predictor variable (EPV) [17, 18]. However, it also suggests that the rule of using ≥10 EPV in Cox models is not clearly defined and can be relaxed. That is, a simulation study suggested that, in comparison to a Cox model with 10–16 EPV, a Cox model with 5–9 EPV provided acceptable results [19]. Therefore, we accepted 5–9 EPV the in the Cox model. In this study, the number of covariates that could be included in the Cox proportional hazards analysis of OS was 15 (i.e., the number of patients who died) divided by 5–9 (result: 1–3) and the number of RFS was 28 (i.e., the number of patients who had a confirmed relapse) divided by 5–9 (result: 3–5). It has been reported that older age, male sex, and advanced pathological stage tend to be associated with a poor prognosis in patients with treated PPC [20, 21]. Therefore, in addition to the gene status variable of interest, we selected age, sex, and pathological stage as confounding factors. Since “elderly” is generally defined as age ≥ 65 years, we set 65 years as the cut-off value for age. We selected the following three factors in the multivariable Cox hazards analysis of OS: gene status (pathogenic mutation [reference: variants of unknown significance (VUSs) or wild type (WT)]) as the explanatory variable; and pathological stage (stage III–IV [reference: stage I–II]) and age (≥65 [reference: < 65]) as confounding factors. Sensitivity analyses were performed by the multivariable Cox hazards analyses of OS in which pathological stages (stage III–IV [reference: stage I–II]) and sex (female [reference: male]), and age (≥65 [reference: < 65]) and sex (female [reference: male]), respectively, were included as confounding factors were performed to assess the robustness of the results of the analysis. On the other hand, we selected the following four factors according to the multivariable Cox hazards analysis of RFS: gene status (pathogenic mutation [reference: VUSs or WT] as the explanatory variable; and pathological stage (stage III–IV [reference: stage I–II]), age (≥65 [reference: < 65] and sex (female [reference: male]) as confounding factors. All statistical analyses were conducted using Easy R (EZR) (Saitama Medical Center, Jichi Medical University, Saitama, Japan), which is a graphical user interface for R (The R Foundation for Statistical Computing, Vienna, Austria). EZR is a modified version of R commander with added biostatistical functions [22]. *P* values of < 0.05 were considered to indicate statistical significance.

## Results

### Patients characteristics

The clinical characteristics of the 55 patients with PPC who underwent complete surgical resection are shown in Table 1. The median age was 65 years (range, 58–72). The majority of the patients were male (75%) and smokers (84%). The pathological stage was mostly advanced (stage II, 42%; stage III, 38%). One case of stage IV with a single brain metastasis was included. In this case, the metastatic brain lesion was treated by gamma knife as oligometastasis followed by complete resection of the primary lunge lesion. Segmentectomy was the least common surgical procedure (2%), while lobectomy was the most common (60%). Advanced cases were also common (pneumonectomy, 9%; lobectomy with complicated resections, 20%). Regarding histological types in PPC, the epithelial component was dominated by adenocarcinoma (71%) and squamous cell carcinoma (20%). The expression of PD-L1 was observed in 95% of cases, and high expression levels (≥50%) were confirmed in 73% of cases. Postoperative relapse was confirmed in 28 of 55 patients (51%) and death after surgery was identified in 15 of 55 patients (27%). The median OS after surgery, which was the median follow-up period in patients who were alive at the end of follow-up, was 893 days (range, 523–1856 days). The follow-up period for OS was identical to that for postoperative relapse. The median follow-up time of postoperative RFS was 562 days (range, 92–1682 days).

**Table 1:** Patient characteristics

Characteristics	Number of patients (*n* = 55)
Demographic	
Age: median (years)	65
Male sex: n (%)	41 (75)
Current/Former Smoker: n (%)	46 (84)
Pathological Stage: n (%)	
I	10 (18)
II	23 (42)
III	21 (38)
IV	1 (2)
Diagnostic procedure: n (%)	
Surgery	54 (98)
Bronchoscopic biopsy	1 (2)
Surgical procedure: n (%)	
Segmentectomy	2 (4)
Lobectomy	33 (60)
Pneumonectomy	5 (9)
Bilobectomy	4 (7)
Lobectomy with combined resection	11 (20)
Epithelial components: n (%)	
Adenocarcinoma	39 (71)
Squamous cell carcinoma	11 (20)
Large cell carcinoma	1 (2)
Large cell neuroendocrine carcinoma	1 (2)
PD-L1 staining: n (%)	
< 1%	3 (5)
1–49%	12 (22)
≥ 50%	40 (73)
Relapse after surgery: n (%)	
No relapse	27 (49)
Relapse after surgery	28 (51)
Survival status at last follow-up: n (%)	
Alive, no evidence of disease	40 (73)
Died of disease	15 (27)
Overall survival after surgery: median (days)	893
Relapse free survival after surgery: median (days)	562

*PD-L1* Programmed cell death-ligand 1

### Genomic alterations in PPC

**Fig. 1:**
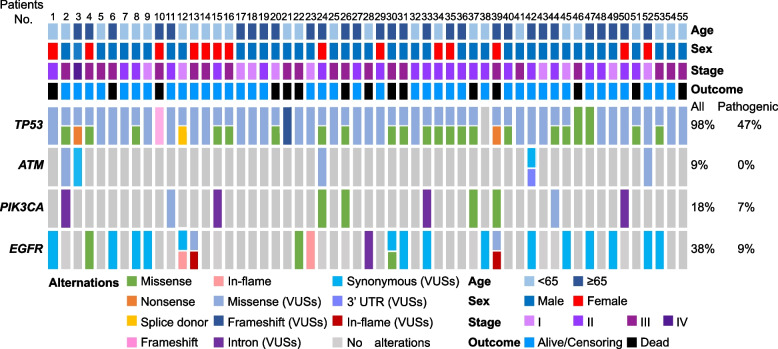
Summary of four genomic mutations of 55 patients with pulmonary pleomorphic carcinoma. All mutations, including variants of unknown significance (VUSs) in *TP53*, *ATM*, *PIK3CA* and *EGFR*, were detected in 54 (98%), 5 (9%), 10 (18%) and 21 (38%) patients, respectively. Pathogenic mutations in *TP53*, *ATM*, *PIK3CA* and *EGFR* were detected in 25 (45%), 0 (0%), 4 (7%) and 5 (9%) patients, respectively

An overview of the mutations of *TP53*, *ATM*, *PIK3CA* and *EGFR* in the primary lesions of 55 patients with PPC is provided in Fig. 1 (detailed information is provided in Supplemental Table S1). *TP53* was the most frequently mutated gene. Among cases with variants of unknown significance (VUSs), *TP53* mutations were detected in 53 (98%) patients. Pathogenic mutations of *TP53* were detected in 26 (47%) patients. The confirmed *ATM* mutations were only VUSs in 5 (9%) patients. *PIK3CA* mutations containing VUSs were detected in 10 (18%) patients. Pathogenic mutations of *PIK3CA* were confirmed in 4 (7%) patients. *EGFR* mutations, including VUSs, were detected in 21 (38%) patients, with 5 (9%) patients only showing pathogenic mutations.

### Overall survival and relapse-free survival after surgery

**Fig. 2:**
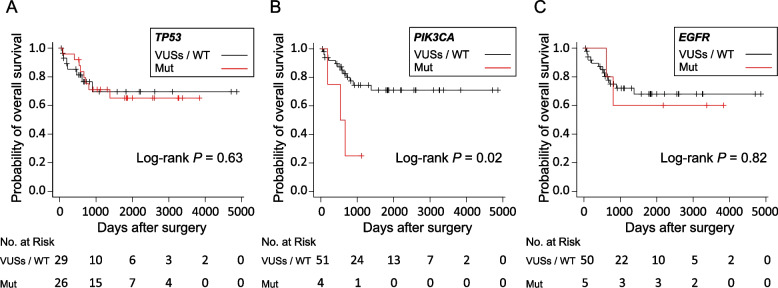
Kaplan-Meier curves showing the probability of overall survival among patients after surgery, (**A**) according to pathogenic mutations of *TP53*, (**B**) according to pathogenic mutations of *PIK3CA*, and (**C**) according to pathogenic mutations of *EGFR*

During a median follow-up period of 893 days after surgery, we confirmed that 15 (27%) patients died because of disease progression. The Kaplan-Meier curve for OS after surgery according to gene mutations—*TP53*, *PIK3CA* and *EGFR* which were detected pathogenic mutations—are shown in Fig. 2. OS after surgery in the *PIK3CA* mutation group was significantly shorter in comparison to the VUSs or WT groups, before adjustment for patient background (*P* < 0.02) (Fig. 2B). The Kaplan-Meier curves for OS after surgery according to the *TP53* mutation and *EGFR* mutation status are shown in Fig. 2A, C. Before adjustment for patient background factors, OS after surgery did not differ among the *TP53* (*P* = 0.63) and *EGFR* (*P* = 0.82) mutation status groups. The Kaplan-Meier curves of RFS after surgery according the *TP53* mutation, *PIK3CA* mutation and *EGFR* mutation status are shown in the supplemental Fig. S1. Before adjusting for patient background factors, RFS after surgery did not differ among the *TP53* (*P* = 0.61), *PIK3CA* (*P* = 0.24) and *EGFR* (*P* = 0.90) mutation status groups.

### Multivariable analyses of factors associated with OS and RFS after surgery according to the gene mutation status

The results of the multivariable Cox proportional hazards analysis of pathogenic mutations associated with OS after surgery are shown in Table 2. A significant difference in OS was observed between the *PIK3CA* mutation group and the VUSs/WT group (adjusted hazard ratio [HR], 4.5; 95% confidence interval [CI] 1.1–18.8) after adjustment for patient background factors (pathological stage and age). On the other hand, OS after surgery did not differ to a statistically significant extent in the *TP53* (adjusted HR, 0.9, 95% CI 0.3–3.1) and *EGFR* mutation groups (adjusted HR, 0.6, 95% CI 0.1–2.8). RFS after surgery did not differ to a statistically significant extent in the *TP53* (adjusted HR, 1.1, 95% CI 0.5–2.5), *PIK3CA* (adjusted HR, 2.2, 95% CI 0.6–8.3) and *EGFR* mutation groups (adjusted HR, 0.8, 95% CI 0.2–2.7) (Supplemental Table S2).

**Table 2:** Cox proportional hazards analysis of OS according to the gene mutation status

	N	Death, N (%)	Unadjusted HR (95% CI), *P*	Adjusted HR^a^ (95% CI), *P*
*TP53*				
VUSs/WT	29	7 (24)	Reference	Reference
Mut	26	8 (31)	1.3 (0.5–3.6), 0.63	0.9 (0.3–3.1), 0.95
*PIK3CA*				
VUSs/WT	51	12 (24)	Reference	Reference
Mut	4	3 (75)	4.3 (1.2–15.7), 0.03	4.5 (1.1–18.8), 0.04
*EGFR*				
VUSs/WT	50	13 (26)	Reference	Reference
Mut	5	2 (40)	1.2 (0.3–5.3), 0.82	0.6 (0.1–2.8), 0.47

*HR* Hazard ratio, *Mut* Pathogenic mutation, *OS* Overall survival, *TP53* gene encoded tumor protein p53, *VUSs* Variants of unknown significance, *WT* Wild type, *PIK3CA* Gene encoded phosphatidylinositol-4,5-bisphosphate 3-kinase catalytic subunit alpha, *EGFR* Gene encoded epidermal growth factor receptor

^a^Adjusted for pathological stage and age

### Sensitivity analyses with different confounding factors in multivariable cox proportional hazards analyses of OS

Sensitivity analyses with modified confounding factors were performed for the *PIK3CA* mutation status, which showed significant differences in the multivariable Cox proportional hazards analysis. For the sensitivity analyses, we used multivariable Cox proportional hazards models with pathological stage and sex, and age and sex, respectively as confounding factors. Significant differences in OS after surgery were observed between the *PIK3CA* mutation group and the VUSs/WT group in both models. The HRs of postoperative OS in cases with *PIK3CA* mutation regarding VUSs/WT were 7.5 (95% CI 1.7–32.4) in the model adjusted for pathological stage and sex and 5.4 (95% CI 1.4–21.7) in the model adjusted for age and sex (Table 3).

**Table 3:** Sensitivity analyses with adjustment for different confounding factors

	Adjusted HR (95% CI), *P*	
	Model 1^**a**^	Model 2^**b**^
*PIK3CA*		
VUSs/WT	Reference	Reference
Mut	7.5 (1.7–32.4), 0.01	5.4 (1.4–21.7), 0.02

*HR* Hazard ratio, *PIK3CA* Gene encoded phosphatidylinositol-4,5-bisphosphate 3-kinase catalytic subunit alpha, *VUSs* Variants of unknown significance, *WT* Wild type, *Mut* Pathogenic mutation

^a^Adjusted for pathological stage and sex

^b^Adjusted for age and sex

### Testing the proportional hazards assumption in cox models

We performed tests of proportional hazards assumption for the Cox models used in the multivariable analyses for OS and RFS. In the tests for proportional hazards assumption for each variable in each Cox model, the *P* values for all variables exceeded 0.05 (Supplemental Table S3 and S4); thus, the proportional hazards assumption was not rejected.

### Efficacy of chemotherapy after postoperative relapse in cases of PPC harboring *PIK3CA* mutation

Of 4 patients with PPC harboring *PIK3CA* pathogenic mutation, one patient (No.24) had no postoperative relapse and was censored at 1114 days after surgery (Table 4). Although the remaining three patients received chemotherapy as an initial treatment, no patients achieved an objective response (Table 4). One patient (No.39) harboring an uncommon *EGFR* mutation was treated with gefitinib after postoperative relapse. However, the patient developed progressive disease in liver metastasis. Two patients (No.37 and No. 39) received immune checkpoint inhibitor (pembrolizumab) treatment after the determination of progressive disease with chemotherapy. Although a partial response was confirmed in patient No.37 2 months after the administration of pembrolizumab, he developed progressive disease with metastasis to the scapula at 4 months after showing a partial response. Patient No.39 showed progressive disease despite the administration of pembrolizumab and was then treated with best supportive care because of poor general condition.

**Table 4:** Clinical course of patients with PPC harboring *PIK3CA* mutation

No.	Sex	Age	Stage	Gene mutation status			PD-L1 TPS%	Chemotherapy		OS days	Outcome
				*TP53*	*ATM*	*EGFR*		1st line	ICI		
24	F	78	II	Mut	VUSs	WT	10	–	–	1114	Censoring
26	M	70	III	VUSs	WT	WT	0	CBDCA+ETP	–	191	Died
37	M	50	II	Mut	WT	WT	40	CBDCA+nab-PTX	Pembro	665	Died
39	F	81	III	Mut	WT	VUSs	70	Gefitinib	Pembro	535	Died

*PPC* Pulmonary pleomorphic carcinoma, *PIK3CA* Gene encoded phosphatidylinositol-4,5-bisphosphate 3-kinase catalytic subunit alpha, *Stage* Pathological stage, *TP53* Gene encoded tumor protein p53, *ATM* Ataxia-telangiectasia mutated gene, *EGFR* Gene encoded epidermal growth factor receptor, *PD-L1* Programmed cell death-ligand 1, *ICI* Immune checkpoint inhibitor, *OS* Overall survival, *VUSs* Variants of unknown significance, *WT* Wild type, *Mut* Mutation, *CBDCA* Carboplatin, *ETP* Etoposide, nab-*PTX* nanoparticle albumin-bound paclitaxel, *Pembro* Pembrolizumab

## Discussion

Two new points that emerged from our study are as follows. First, although *TP53* mutations were most frequent in PPC, *TP53* mutations were not significantly associated with postoperative OS. Second, although the frequency of *PIK3CA* mutations in PPC was only a few percent, the postoperative OS of patients with *PIK3CA* mutations was significantly shorter. To our knowledge, this is the first report of a relatively large (*n* = 55) study evaluating the association between genetic variants in PPC and the clinical prognosis after complete resection.

Regarding the first point (i.e., the association between PPC and *TP53* mutations), we found a high frequency of *TP53* mutations in our PPC cohort (26/55). *TP53* mutation has been reported to be the most frequently mutated gene in lung cancers of all histological types [23]. Resistance to radiotherapy and chemotherapy due to *TP53* mutations has also been suggested to be associated with a poor prognosis [24]. However, in our cohort, no significant association was found between *TP53* mutations in PPC and the postoperative prognosis. Therefore, the prognostic impact of *TP53* mutations in PPC may differ from that in other lung cancers; further studies are needed to clarify the association between *TP53* mutations and the prognosis in patients with PPC. *ATM*, similarly to *TP53*, is a gene known to be involved in the repair pathway of damaged DNA. It has been reported that mutations in *ATM* are found in approximately 10% of lung cancers [25]. The frequency of *ATM* mutations in our PCC cohort was 9% (5/55), which is in line with previous studies [9]. On the other hand, no pathogenic mutations of *ATM* were detected in our cohort (0/55). This result is consistent with a previous report about PPC [23]. Pathogenic mutations of *ATM* may tend to be relatively rare in PPC.

Regarding the second point (i.e., the association between PPC and *PIK3CA* mutations), a previous study of PPC indicated that the frequency of *PIK3CA* mutations in PPC was 20% for variants including VUSs and 10% for pathogenic mutations alone [23]. On the other hand, the frequency of *PIK3CA* mutations in NSCLC is reported to be as low as 1.8–3% [26, 27]. In our PPC cohort, the frequency of *PIK3CA* mutations was 18% (10/55) for variants including VUSs and 7% (4/55) for pathogenic mutations alone, which was largely consistent with the previous reports [9, 23]. It is suggested that the frequency of *PIK3CA* mutations in PPC tends to be higher in comparison to that of *PIK3CA* mutations in NSCLC. Approximately 70% of the cases of PPC in our cohort contained adenocarcinoma as an epithelial component. Although it has been reported that 80% of lung adenocarcinoma with *PIK3CA* mutations is associated with *EGFR* mutations [28], no cases with pathogenic co-mutation of *PIK3CA* and *EGFR* were detected in our PPC cohort. That is, *PIK3CA* and *EGFR* mutations may be functionally independent in PPC. In addition, we found that *EGFR* mutations have no significant effect on the postoperative prognosis of PPC, whereas *PIK3CA* mutations were significantly associated with the postoperative OS. However, the association between *PIK3CA* mutations and RFS after surgery was not confirmed. These results suggest a significant contribution of *PIK3CA* mutations to the resistance of postoperative treatment in PPC.

Three of the four patients with pathogenic *PIK3CA* mutations relapsed after surgery and received chemotherapy; however, all of these patients died, which suggested resistance to chemotherapy. PPC has been reported to be associated with the high expression of PD-L1 [21], and 75% of the patients in our cohort showed high expression levels. The use of immune checkpoint inhibitors (ICIs) for PPC has been reported, and is suggested to be effective [29]. The outcomes of our *PIK3CA* mutation-positive PPC cases that were treated with chemotherapy and ICIs may be biologically explainable. No details are known about the mechanisms by which PPC develop resistance to chemotherapy. However, the mechanism of chemotherapy resistance in small cell lung cancer, which is characterized by many abnormalities in signaling pathways involving *PIK3CA* (PI3K/Protein kinase B [Akt]/the mammalian target of rapamycin [mTOR] pathway), may be informative. It has been reported that small cell lung cancer may be associated with chemotherapy resistance due to a phenotypic transition from suspension to an adhesion growth pattern caused by the activation of the PI3K/Akt/mTOR pathway [30]. *PIK3CA* mutation-positive PPC may have a similar mechanism. Our patient No.26, who was diagnosed before ICIs were covered by insurance, showed chemotherapy resistance. Inhibitors of the PI3K/Akt/mTOR pathway may be effective against PPC with *PIK3CA* mutations, because chemotherapy-resistant cell lines are reported to be sensitive to PI3K inhibitors [31]. Cases No.37 and 39 were resistant to 1st-line chemotherapy and subsequently received pembrolizumab. However, no significant immunotherapy response was observed. A mechanism of immunotherapy resistance in gastric cancer has been reported, in which activation of the PI3K/Akt/mTOR pathway increases the production of free fatty acids, which are taken up more efficiently by regulatory T cells than by effector T cells, resulting in an increase in regulatory T cells in the tumor [32, 33]. Similar changes in the immune environment occur in PPC with *PIK3CA* mutations, which may explain why ICIs are not fully effective for PPC harboring *PIK3CA* mutations. On the other hand, it has been reported that in NSCLC, patients with both *TP53* and *ATM* mutations showed a higher response to treatment for ICIs due to having a significantly higher tumor mutation burden in comparison to groups with either mutation alone or without mutation [34]. Cases No.37 and 39 were *TP53* mutation-positive and *ATM* mutation-negative, which may have limited the therapeutic effect of ICIs. Therefore, the use of PI3K/Akt/mTOR pathway inhibitors may be effective in PPC with *PIK3CA* mutations as a strategy to enhance the efficacy of ICIs. Precision medicine, in which combinations of *PIK3CA*, *TP53* and *ATM* mutations in PPC are identified and PI3K/Akt/mTOR pathway inhibitors or ICIs are administered according to the mutations pattern, may become an effective treatment for PPC with *PIK3CA* mutations in the future.

The present study was associated several limitations. First, the number of confounders that could be included in the multivariable Cox proportional hazards model was limited by the small number of cases due to the rarity of PPC, making it impossible to include many factors in that model at one time. Therefore, the robustness of the results was assessed using a sensitivity analysis to vary the combination of factors included in the multivariable Cox proportional hazards model. Although ≥10 EPV is recommended for a Cox proportional hazards model, 5–9 EPV was accepted for our study based on reports that have demonstrated that it is possible to relax this constraint [19]. However, further studies are required to determine whether similar results can be obtained with sample sizes that satisfy the standard condition of ≥10 EPV. Second, because most cases of PPC are divided into epithelial and sarcomatoid components, it is strictly necessary to analyze the genomic DNAs from each component. However, the difference in the PPC component may not be very important, since it has been reported that approximately 70% of the epithelial and sarcomatoid components share the same driver gene mutation [9]. Third, the molecular mechanism underlying short postoperative overall survival and chemotherapy resistance, including ICIs of PPC harboring *PIK3CA* mutation is unclear. Further studies are needed in order to confirm this. Fourth, only four patients had *PIK3CA* mutations, and their survival probability was compared to that of 51 patients without *PIK3CA* mutations. Although the multivariable analysis confirmed a survival difference, the possibility that this difference occurred by chance cannot be ruled out. Therefore, further confirmatory studies are warranted.

## Conclusion

We evaluated the relationship between gene mutations (*TP53*, *ATM*, *PIK3CA*, *EGFR*) and overall survival after surgery in PPC, and found that patients with *PIK3CA* mutations had significantly shorter postoperative overall survival. To our knowledge, this is the first report of this finding. *PIK3CA* mutations are prognostic markers for PPC, and inhibition of the *PIK3CA*-related pathway may contribute to improving the prognosis of patients with PPC, including improving the efficacy of ICI therapy.

## Supplementary Information

**Additional file 1: Supplemental Table S1.** NGS results for 55 patients with pulmonary pleomorphic carcinoma.

**Additional file 2: Supplemental Fig. S1.** The Kaplan-Meier curves of RFS after surgery according the *TP53* mutation (A), *PIK3CA* mutation (B) and *EGFR* mutation (C) status.

**Additional file 3: Supplemental Table S2.** The Cox proportional hazards analysis of RFS according to the gene mutation status.

**Additional file 4: Supplemental Table S3.** Testing the proportional hazards assumption in Cox models for OS.

**Additional file 5: Supplemental Table S4.** Testing the proportional hazards assumption in Cox models for RFS.

## Data Availability

The datasets generated and analyzed during the current study are available in the DNA Data Bank of Japan (DDBJ) Sequenced Read Archive repository (http://www.ddbj.nig.ac.jp/index.html) under accession number DRA014590.

## Abbreviations

*ATM*: Ataxia-telangiectasia mutated gene
*EGFR*: Epidermal Growth Factor Receptor gene
NSCLC: Non-Small Cell Lung Cancer
HR: Hazard Ratio
*TP53*: tumor protein p53 gene
*PIK3CA*: phosphatidylinositol-4,5-bisphosphate 3-kinase catalytic subunit alpha gene
PPC: Pulmonary Pleomorphic Carcinoma
PD-L1: programmed cell death-ligand 1
OS: Overall Survival
VUSs: variants of unknown significance
WT: Wild Type
