# Supplementary material for: PIK3CA mutations associated with a poor postoperative prognosis in patients with pulmonary pleomorphic carcinoma: a retrospective cohort study
Source: BMC Cancer. 2022 Oct 15;22:1066. doi: 10.1186/s12885-022-10176-4 (PMC9571475; doi:10.1186/s12885-022-10176-4)
Supplement: Supplementary file 2 — Additional file 2: Supplemental Fig. S1. The Kaplan-Meier curves of RFS after surgery according the TP53 mutation (A), PIK3CA mutation (B) and EGFR mutation (C) status. [file 12885_2022_10176_MOESM2_ESM.pdf]

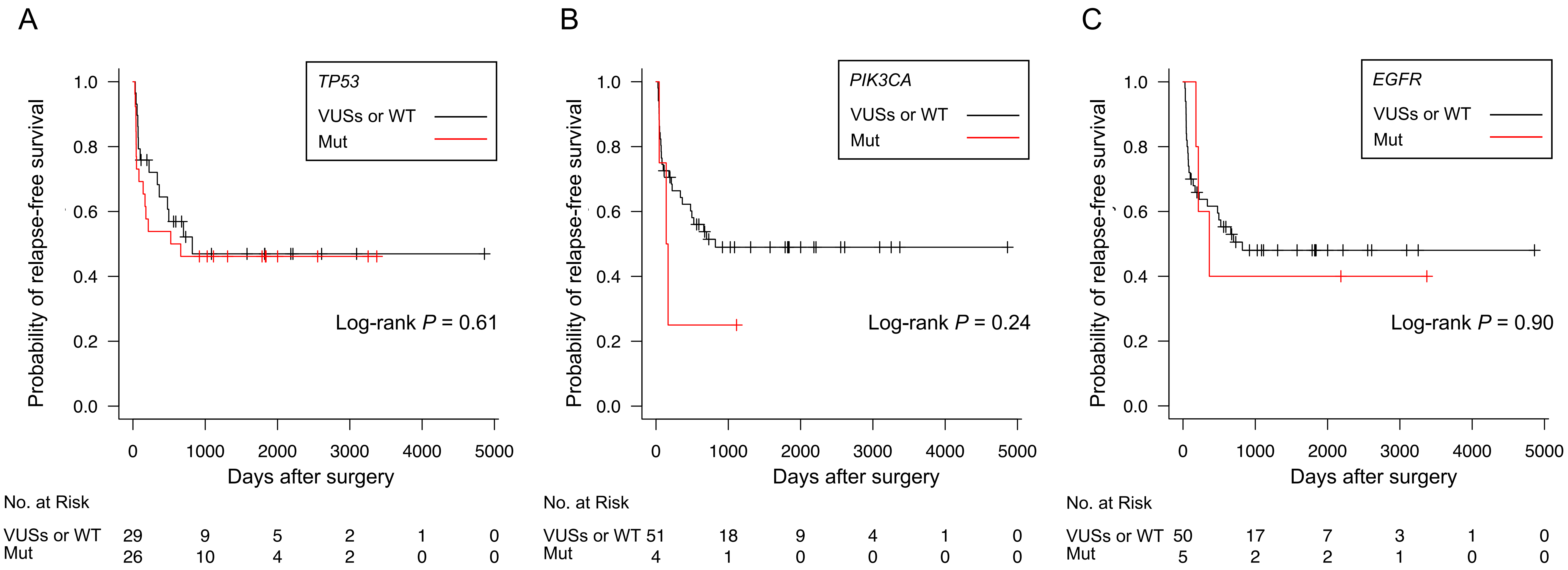

Supplemental Figure S1: The Kaplan-Meier curves of RFS after surgery according the *TP53* mutation (A), *PIK3CA* mutation (B) and *EGFR* mutation (C) status.
