# Supplementary material for: PIK3CA mutations associated with a poor postoperative prognosis in patients with pulmonary pleomorphic carcinoma: a retrospective cohort study
Source: BMC Cancer. 2022 Oct 15;22:1066. doi: 10.1186/s12885-022-10176-4 (PMC9571475; doi:10.1186/s12885-022-10176-4)
Supplement: Supplementary file 3 — Additional file 3: Supplemental Table S2. The Cox proportional hazards analysis of RFS according to the gene mutation status. [file 12885_2022_10176_MOESM3_ESM.docx]

Supplemental Table S2. The Cox proportional hazards analysis of RFS according to the gene mutation status.

|  | N | Relapse, N (%) | Unadjusted HR (95% CI), *P* | Adjusted HR^a^ (95% CI), *P* |
| --- | --- | --- | --- | --- |
| *TP53* | | | | |
| VUSs/WT | 29 | 14 (48) | Reference | Reference |
| Mut | 26 | 14 (54) | 1.2 (0.6–2.5), 0.61 | 1.1 (0.5–2.5), 0.83 |
| *PIK3CA* | | | | |
| VUSs/WT | 51 | 25 (49) | Reference | Reference |
| Mut | 4 | 3 (75) | 2.0 (0.6–6.7), 0.25 | 2.2 (0.6–8.3), 0.26 |
| *EGFR* | | | | |
| VUSs/WT | 50 | 25 (50) | Reference | Reference |
| Mut | 5 | 3 (60) | 1.1 (0.3–3.6), 0.90 | 0.8 (0.2–2.7), 0.68 |

*HR* hazard ratio, *Mut* pathogenic mutation, *RFS* relapse-free survival, *PIK3CA* gene encoded phosphatidylinositol-4,5-bisphosphate 3-kinase catalytic subunit alpha, *VUSs* variants of unknown significance, *WT* wild type.

^a^ Adjusted for pathological stage, age and sex.
