# Supplementary material for: PIK3CA mutations associated with a poor postoperative prognosis in patients with pulmonary pleomorphic carcinoma: a retrospective cohort study
Source: BMC Cancer. 2022 Oct 15;22:1066. doi: 10.1186/s12885-022-10176-4 (PMC9571475; doi:10.1186/s12885-022-10176-4)
Supplement: Supplementary file 4 — Additional file 4: Supplemental Table S3. Testing the proportional hazards assumption in Cox models for OS. [file 12885_2022_10176_MOESM4_ESM.docx]

Supplemental Table S3. Testing the proportional hazards assumption in Cox models for OS.

| Explanatory variables in the Cox models | *P* |
| --- | --- |
| *TP53* mutation status, pathological stage and age | |
| *TP53* Mut (reference: VUSs/WT) | 0.24 |
| Pathological stage: stage III–IV (reference: stage I–II) | 0.25 |
| Age: ≥65 (reference: <65) | 0.18 |
| *TP53* mutation status, pathological stage and sex | |
| *TP53* Mut (reference: VUSs/WT) | 0.33 |
| Pathological stage: stage III–IV (reference: stage I–II) | 0.50 |
| Sex: female (reference: male) | 0.31 |
| *TP53* mutation status, age and sex | |
| *TP53* Mut (reference: VUSs/WT) | 0.31 |
| Age: ≥65 (reference: <65) | 0.18 |
| Sex: female (reference: male) | 0.39 |
| *PIK3CA* mutation status, pathological stage and age | |
| *PIK3CA* Mut (reference: VUSs/WT) | 0.16 |
| Pathological stage: stage III–IV (reference: stage I–II) | 0.44 |
| Age: ≥65 (reference: <65) | 0.15 |
| *PIK3CA* mutation status, pathological stage and sex | |
| *PIK3CA* Mut (reference: VUSs/WT) | 0.16 |
| Pathological stage: stage III–IV (reference: stage I–II) | 0.73 |
| Sex: female (reference: male) | 0.27 |
| *PIK3CA* mutation status, age and sex | |
| *PIK3CA* Mut (reference: VUSs/WT) | 0.40 |
| Age: ≥65 (reference: <65) | 0.27 |
| Sex: female (reference: male) | 0.47 |
| *EGFR* mutation status, pathological stage and age | |
| *EGFR* Mut (reference: VUSs/WT) | 0.09 |
| Pathological stage: stage III–IV (reference: stage I–II) | 0.13 |
| Age: ≥65 (reference: <65) | 0.06 |
| *EGFR* mutation status, pathological stage and sex | |
| *EGFR* Mut (reference: VUSs/WT) | 0.18 |
| Pathological stage: stage III–IV (reference: stage I–II) | 0.40 |
| Sex: female (reference: male) | 0.35 |
| *EGFR* mutation status, age and sex | |
| *EGFR* Mut (reference: VUSs/WT) | 0.24 |
| Age: ≥65 (reference: <65) | 0.19 |
| Sex: female (reference: male) | 0.53 |

*TP53* gene encoded tumor protein p53, *Mut* pathogenic mutation, *VUSs* variants of unknown significance, *WT* wild type, *PIK3CA* gene encoded phosphatidylinositol-4,5-bisphosphate 3-kinase catalytic subunit alpha, *EGFR* gene encoded epidermal growth factor receptor.
