# Supplementary material for: PIK3CA mutations associated with a poor postoperative prognosis in patients with pulmonary pleomorphic carcinoma: a retrospective cohort study
Source: BMC Cancer. 2022 Oct 15;22:1066. doi: 10.1186/s12885-022-10176-4 (PMC9571475; doi:10.1186/s12885-022-10176-4)
Supplement: Supplementary file 5 — Additional file 5: Supplemental Table S4. Testing the proportional hazards assumption in Cox models for RFS. [file 12885_2022_10176_MOESM5_ESM.docx]

Supplemental Table S4. Testing the proportional hazards assumption in Cox models for RFS.

| Explanatory variables in the Cox models | *P* |
| --- | --- |
| *TP53* mutation status, pathological stage, age and sex | |
| *TP53* Mut (reference: VUSs/WT) | 0.08 |
| Pathological stage: stage III–IV (reference: stage I–II) | 0.15 |
| Age: ≥65 (reference: <65) | 0.40 |
| Sex: female (reference: male) | 0.91 |
| *PIK3CA* mutation status, pathological stage, age and sex | |
| *PIK3CA* Mut (reference: VUSs/WT) | 0.43 |
| Pathological stage: stage III–IV (reference: stage I–II) | 0.16 |
| Age: ≥65 (reference: <65) | 0.91 |
| Sex: female (reference: male) | 0.63 |
| *EGFR* mutation status, pathological stage, age and sex | |
| *EGFR* Mut (reference: VUSs/WT) | 0.14 |
| Pathological stage: stage III–IV (reference: stage I–II) | 0.30 |
| Age: ≥65 (reference: <65) | 0.97 |
| Sex: female (reference: male) | 0.83 |

*TP53* gene encoded tumor protein p53, *Mut* pathogenic mutation, *VUSs* variants of unknown significance, *WT* wild type, *PIK3CA* gene encoded phosphatidylinositol-4,5-bisphosphate 3-kinase catalytic subunit alpha, *EGFR* gene encoded epidermal growth factor receptor.
